# When rules stick too well: overgeneralization of physical and psychological noun phrase attachment in L2 learning of Japanese sound-symbolic words

**DOI:** 10.3389/fpsyg.2026.1906329

**Published:** 2026-10-26

**Authors:** Yajing Feng, Katsuo Tamaoka

**Affiliations:** 1 School of International Studies, Hangzhou Normal University, Hangzhou, China; 2 School of Foreign Studies, Shanghai University, Shanghai, China; 3 Graduate School of Humanities, Nagoya University, Nagoya, Japan

**Keywords:** Japanese sound-symbolic words, L2 grammatical proficiency, NP attachment, overgeneralization, psychomimes

## Abstract

**Introduction:**

This study investigates the overgeneralization of noun phrase (NP) attachment rules in the L2 acquisition of Japanese sound-symbolic words, focusing on psychomime verbs that describe physical and psychological sensations.

**Methods:**

An experiment using an NP attachment judgement task was conducted with 88 Chinese-speaking learners of Japanese (CSLJs) and 76 Japanese native speakers (JNSs) across NP attachment (Class 1), suppression (Class 3), and free conditions (Class 2).

**Results:**

The results showed that overgeneralization was category-specific. CSLJs performed with high accuracy on Class 1 items, where both Chinese and Japanese require NP attachment, but struggled with Class 3 items, where Japanese requires NP suppression. Class 2, occupying an intermediate position between concrete bodily sensation and abstract psychological states, imposed the greatest cognitive demands and emerged as the strongest predictor of learners’ overall NP attachment preference. This overgeneralization was shaped by both L1 transfer and L2 grammatical proficiency. Learners initially relied on L1-derived processing strategies due to properties of Mandarin, yet this reliance diminished with increasing proficiency, indicating that L1 transfer is a dynamic rather than static constraint. A threshold effect was observed, with only high-proficiency learners able to suppress the overgeneralized NP attachment rule and perform in a manner similar to JNSs, whereas middle- and low-proficiency learners continued to overgeneralize. Proficiency, when entered as a continuous variable, did not significantly predict NP attachment preference, confirming that proficiency’s influence is non-linear.

**Discussion:**

These findings collectively demonstrate that the resolution of overgeneralization is structure-specific and depends on the interaction between L2 proficiency, structural markedness, and L1-L2 overlap. The study contributes to a more general understanding of L2 development by elucidating the dynamic interaction between syntactic form and semantic interpretation at the syntax–semantics interface.

## 1 Introduction

Japanese sound-symbolic words are pervasive in Japanese writing, reading, and daily spoken discourse. They fall into three major categories: phonomimes that mimic sounds, phenomimes that convey visual and tactile events, and psychomimes that encode emotional and physical sensations (Hamano, 1998; Hasada, 1998). The research has shown consistently that even proficient L2 Japanese learners with different linguistic backgrounds—including Chinese, English, Korean, Dutch, and Russian—encounter substantial difficulties in acquiring Japanese sound-symbolic words, which is in stark contrast to the effortless acquisition observed in Japanese children (Iwasaki, 2017; Yoshioka, 2017; Feng and Tamaoka, 2018, 2026; Manuylova, 2020; Li, 2021). Among the three subtypes, psychomimes pose the greatest learning challenges because of their relatively low lexical iconicity (Akita, 2009), which forces L2 learners to rely heavily on abstract grammatical structures, particularly NP attachment rules, for semantic interpretation. This situation renders learners vulnerable to form–meaning mapping failures regarding NP attachment rules in psychomime constructions.

Notably, such grammatical form–meaning mapping difficulties are not unique to the acquisition of Japanese sound-symbolic words. Instead, they reflect universal challenges in second language acquisition (SLA), including grammatical rule overgeneralization and the incomplete internalization of form–meaning associations in the mental lexicon (e.g., DeKeyser, 2000; DeKeyser, 2015; Ellis, 2006). In this context, the present study investigates systematically NP attachment rules associated with physical and psychological meaning in Japanese psychomime verb sentences.

It aims to explore how syntactic grammatical forms shape L2 learners’ semantic acquisition of psychomimes and to uncover the general mechanisms governing the acquisition and mastery of such lexical items. Specifically, this study examines how syntactic NP attachment rules interact with the semantic interpretation of different psychomime categories, an issue at the syntax–semantics interface, and investigates whether a similar overgeneralization pattern can be observed among CSLJs, and how L2 grammatical proficiency modulates this pattern.

## 2 Literature review

### 2.1 Features and L1 acquisition of Japanese sound-symbolic words

Sound-symbolic words simulating objects, actions and phenomenon sounds are widely termed mimetics, ideophones, or expressives in linguistics and are formally defined as lexical items encoding sensory imagery (Doke, 1935; Fivaz, 1963; Diffloth, 1972; Martin, 1975; Mester and Itô, 1989; Schourup and Tamori, 1992; Wiltshire, 1999; Dingemanse, 2011, 2012). In Japanese, such items are referred to as オノマトペ (*onomatope*), derived from the English term onomatopoeia (Chang, 1990). Unlike most languages that feature only sound-imitating lexicons, Japanese sound-symbolic words fall into three distinct categories: phonomimes for natural and animal sounds, phenomimes for external states and actions, and psychomimes for internal emotional experiences (Hasada, 1994, 1998; Kakehi et al., 1996). Such words have rhythmic phonetic properties and strong expressiveness—allowing vivid descriptions of external sounds, physical states and inner feelings—and are widely used in daily communication, literature, news, advertisements and manga. These words also show systematic phonetic and symbolic features at segmental and suprasegmental levels (Hamano, 1998; Ando et al., 2021). Related psychological experiments have shown that they connect more closely with human perceptual experiences and carry unique sound-symbolic traits that general vocabulary words lack (Osaka et al., 2004; Hashimoto et al., 2006; Kanero et al., 2014; Wong et al., 2022). For instance, the classic *bouba*-*kiki* effect demonstrates that even novel pseudowords are systematically associated with particular shapes based on their sound properties, with rounded sounds typically linked to round shapes and sharp sounds to spiky shapes (Kasap and Ünsal, 2024).

In addition to their linguistic characteristics, Japanese sound-symbolic words play a vital role in first language acquisition (FLA) for young children. Infant babbling can even be regarded as an early form of these words (Osaka, 1999). Studies have shown that compared with caregivers from other regions, Japanese mothers use far more sound-symbolic words to communicate with infants (Toda et al., 1990; Fernald and Morikawa, 1993). Such words are frequently applied in motherese and preschool teaching to describe actions (Kondo and Watanabe, 2008). The frequency of these words in maternal language input is correlated positively with children’s corresponding outputs, and children who use more sound-symbolic words tend to have better speech fluency and richer vocabularies (Ogino and Kobayashi, 1999). Since these words are commonly seen in picture books, nursery rhymes and fairy tales, they are among the first lexical items acquired by Japanese toddlers (Takahashi, 2003; Kasai, 2012; Fukada, 2013). Furthermore, early command of Japanese sound-symbolic words facilitates the learning of general vocabulary, laying a firm foundation for children’s overall language development (Imai et al., 2008, 2015; Imai and Kita, 2014).

### 2.2 L2 Acquisition of Japanese sound-symbolic words

The above findings raise a critical research question: whereas Japanese sound-symbolic words are acquired effortlessly by native children in FLA, can L2 learners achieve comparable mastery of these items? In contrast to FLA evidence, the acquisition of Japanese sound-symbolic words poses far greater challenges for L2 learners than general vocabulary learning does (Iwasaki et al., 2017). Empirical studies confirm that even advanced L2 Japanese learners struggle with the comprehension and production of such words, primarily because of their distinctive grammatical properties (Hamano, 1998; Feng and Tamaoka, 2018). These lexical items feature a high degree of grammatical complexity and structural flexibility (Tamori and Schourup, 1999). Specifically, phonomimes typically function as adverbs, phenomimes can serve as adverbs or *suru* light verbs, and psychomimes predominantly appear as *suru* light verbs. Compared with phonomimes and phenomimes, psychomimes exhibit lower lexical iconicity, which further hinders L2 learners’ semantic acquisition (Akita, 2009).

Although the research has documented thoroughly the difficulty of learning Japanese sound-symbolic words in L2 contexts, few studies have focused specifically on psychomime acquisition, leaving a notable research gap. Semantically, Japanese psychomimes fall into two core types: those encoding concrete physical sensations and those conveying abstract psychological states whose syntactic structures are closely tied to semantic abstractness (Akita, 2009). When combined with *suru* verbs, psychomime constructions are strictly constrained by physical and psychological NP attachment rules. In this context, this study examines NP attachment rules in psychomime verb sentences and explores how syntactic properties shape L2 learners’ semantic acquisition of psychomimes. These findings have pedagogical implications for L2 acquisition of Japanese sound-symbolic words and advance the relevant theoretical research in SLA.

### 2.3 NP attachment rules of psychomime verb types

On the basis of NP markers, Kageyama (2007) grouped Japanese psychomime verbs into two categories: those for physical sensations requiring NP markers and those for psychological states without NP markers. Akita (2010) further classified them into bodily sensation, somatopsych and visuopsych subtypes through semantic, syntactic and morphological analyses (see (1) for details and examples).

(1) Classification of psychomime verbs (Akita, 2010)

(1-1) Class 1: mimetics for bodily sensation

a. *Sato-wa atama-ga **zukizuki** si-te i-ru.*

S.-Top head-NOM MIM do-CONJ be-NPST

‘Sato has a throbbing pain in his head.’

*b. *Sato-wa **zukizuki** si-te i-ru*

S.-Top MIM do-CONJ be-NPST

‘Sato has a throbbing pain.’

(1-2) Class 2: somatopsych-mimetics

a. *Sato-wa mune-ga **wakuwaku** si-te i-ru.*

S.-Top chest-NOM MIM do-CONJ be-NPST

‘Sato is excited with chest heaving.’

b. *Sato-wa **wakuwaku** si-te i-ru*.

S.-Top MIM do-CONJ be-NPST

‘Sato is excited.’

(1-3) Class 3: visuopsych-mimetics

*a. *Sato-wa kokoro-ga **kuyokuyo** si-te i-ru*.

S.-Top heart-NOM MIM do-CONJ be-NPST

‘Sato’s heart is mopey.’

b. *Sato-wa **kuyokuyo** si-te i-ru*.

S.-Top MIM do-CONJ be-NPST

‘Sato is mopey.’

Note: The example sentences marked with ‘*’ represent sentences that are unnatural and not fluent; ‘*si-te i-ru*’ is the present progressive tense of the *suru* verb in Japanese.

Class 1 words describe involuntary physical sensations tied to specific body parts. As shown in (1–1), *zukizuki* (‘throbbing pain’) collocates necessarily with the NP *atama-ga* (‘head’). Class 2 somatopsych items convey emotions via physical reactions, allowing optional attachment of the NP *mune-ga* (‘chest’), as illustrated in (1–2). Class 3 visuopsych words express inner feelings exclusively. As (1–3) demonstrates, *kuyokuyo* (‘feeling gloomy’) cannot co-occur with the NP *kokoro-ga* (‘heart’).

Apart from syntactic variations, Class 1 and Class 3 psychomimes differ fundamentally in lexical conceptual structure (Kageyama, 2006, 2007). Their structures with the *suru* light verb are demonstrated in (2). Class 1 examples such as *zukizuki* (‘throbbing pain’) describe abnormal sensations in specific body parts. Since the body-part subject is beyond voluntary control, it cannot be omitted. For Class 3 items such as *gakkari* (‘feeling dejected’), the agent subject undergoes a psychological state caused by an external element.

(2) Lexical conceptual structure of Class 1 and Class 3 (Kageyama, 2006)

(2-1) Lexical conceptual structure of Class 1: [Spkˆ COGNIZE [Spk’s [- BODY-PART] MOVE <Manner α>]]

E.g.: *Atama-ga **zukizuki** si-te i-ru*.

head-NOM MIM do-CONJ be-NPST

‘The head is having a throbbing pain.’

(2-2) exical conceptual structure of Class 3: [x EXPERIENCE [BECOME [x BE AT [- PSYCH.STATE <Manner α> ABOUT -z]]]]

E.g.: *Watasi-wa siken-no kekka-ni **gakkari** si-ta*.

I-Top exam-GEN result-DAT MIM do-PST

‘I was disappointed with the results of the exam.’

Kageyama (1996, 2006) also noted the universal correspondence between lexical conceptual structure and syntactic structure, as shown in (3). When the central meaning of a lexical concept is [MOVE], [BECOME], or [BE], the element, which functions as the subject in the syntactic structure, serves as an internal argument in the verb’s argument structure and appears as the object or a subject equivalent to the object. Conversely, when [ACT], [CONTROL], [EXPERIENCE], or [CAUSE] are the central meanings of the lexical conceptual structure, the element [x], which serves as the sentence subject, is the external argument in the verb’s argument structure.

(3) The universal correspondence between lexical conceptual structure and syntactic structure (Kageyama, 1996, 2006)

(3–1) Lexical conceptual structure: [y MOVE/BECOME/BE].

Syntactic structure: y serves as the internal argument.

(3–2) Lexical conceptual structure: [x ACT/CONTROL/EXPERIENCE/CAUSE].

Syntactic structure: x serves as the external argument.

On the basis of the above research, Class 1 physical verbs and Class 3 psychological verbs show obvious syntactic differences. We reanalyzed examples (1–1a) and (1–3b), with the results presented in the tree diagrams (see Figure 1). With respect to Class 1, physical NPs require mandatory marking, defined here as mandatory NP attachment. Class 2 allows optional marking of physical or psychological NPs, termed optional NP attachment. For Class 3, psychological NPs cannot be marked, which we refer to as NP suppression.

**Figure 1:**
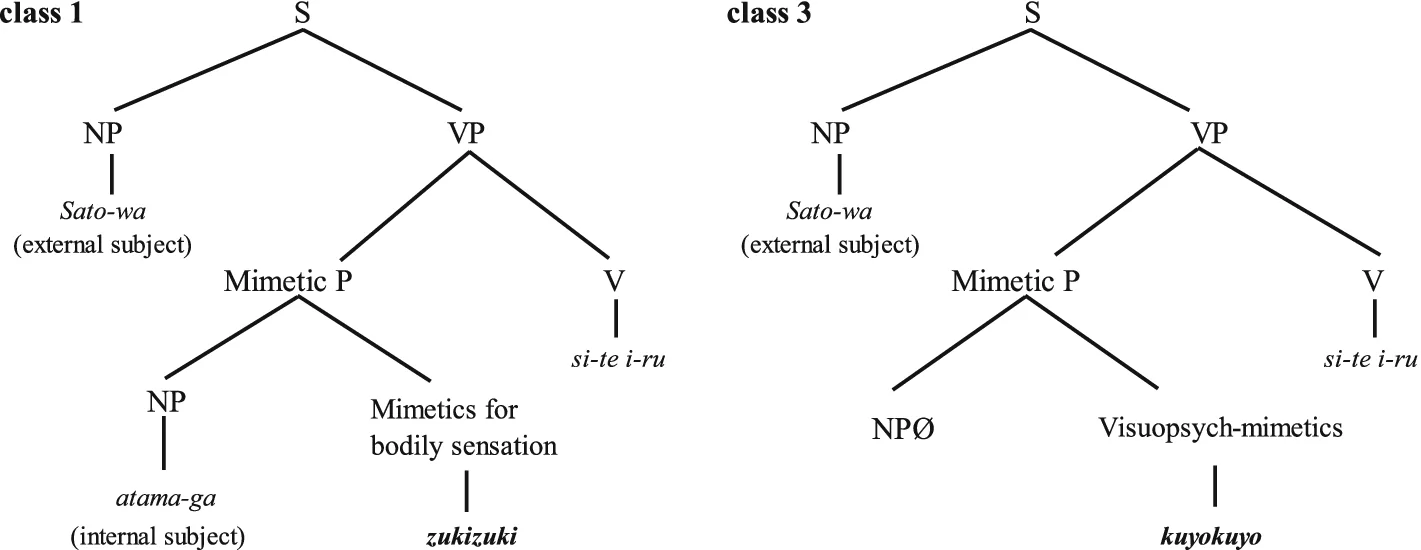
Tree diagrams of Class 1 *zukizuki* and Class 3 *kuyokuyo.*

### 2.4 Overgeneralization in L2 syntactic acquisition

#### 2.4.1 Definition of overgeneralization

Overgeneralization has long been regarded as one of the most characteristic developmental phenomena in language acquisition (Brown, 1973; Bowerman, 1982; Braine and Brooks, 1995). Broadly speaking, overgeneralization occurs when learners extend a learned pattern beyond its legitimate scope. Overgeneralization has been extensively investigated in FLA, where a rich empirical foundation has been established (e.g., Brooks et al., 1999; Ambridge, 2013; Ambridge et al., 2014, 2018; Bidgood et al., 2021; Ringstad and Kush, 2021; Hržica et al., 2023). The phenomenon has also received considerable attention in SLA research (e.g., Inagaki, 1997; Tamburelli, 2008; Moloi, 2013; Xiang et al., 2024; Wei et al., 2026), though many theoretical and empirical questions remain open.

Overgeneralization falls into two primary types: rule overgeneralization and semantic overextension. Rule overgeneralization refers to the extension of a grammatical rule to contexts where it does not apply, whereas semantic overextension involves the inappropriate expansion of a lexical item’s semantic range. Ambridge et al. (2013) distinguished overgeneralizations at the lexical level (e.g., overextending *doggie to bears and horses) from those at the morphological (e.g., *unsqueeze, *sitted) and syntactic levels (e.g., *Don’t giggle me), suggesting that the former involves semantic overextension while the latter reflects rule overgeneralization. This latter type, rule overgeneralization, has been documented across various grammatical domains, including morphological inflection (Brown, 1973; Kuczaj, 1977; Larsen-Freeman, 2010), syntactic structures (Kuribara, 2004; White, 1989), and lexical-syntactic mappings (Ard and Gass, 1987; Pinker, 1984, 1989; Juffs, 1996; Helms-Park, 2002; Xiang et al., 2024; Kawaguchi, 2013).

The present study focuses on rule overgeneralization in the domain of NP attachment with Japanese psychomimes. Although the classification of psychomimes adopted here is grounded in lexical semantics (Akita, 2010; Kageyama, 1996, 2006), the overgeneralization phenomenon under investigation is not semantic overextension (the inappropriate expansion of a word’s meaning) but rather the application of NP attachment rules to syntactic contexts where they are grammatically illicit. In this study, “overgeneralization” refers exclusively to rule overgeneralization unless otherwise specified.

While acknowledging this conceptual distinction, we also recognize that rule overgeneralization and semantic overextension are not entirely separate phenomena. Rather, inaccurate semantic representations of verbs may serve as the underlying cause of syntactic overgeneralization, revealing an intrinsic connection between the two (Ard and Gass, 1987; Pinker, 1984, 1989; Juffs, 1996; Helms-Park, 2002; Kawaguchi, 2013). Kawaguchi (2013) argued that lexical ability, particularly verb learning, is closely related to syntactic development in SLA, observing that grammatical constructions are lexically restricted and that more complex verbs are required for complex sentence production. Helms-Park (2002) further observed that overgeneralization errors are not simply a matter of syntactic rule misapplication. They often reflect learners’ insufficient awareness of the semantic boundaries that constrain verb class membership. Substantial overgeneralization errors with directional motion verbs were observed across all proficiency levels, including among advanced learners, suggesting that inaccurate or underspecified semantic representations of verb classes can persist and systematically trigger syntactic overgeneralization. This lends further support to the view that rule overgeneralization and semantic overextension are intrinsically linked. Imprecise semantic knowledge of a verb class may lead learners to overextend syntactic rules to verbs that, semantically, do not license them.

#### 2.4.2 Theoretical frameworks accounting for overgeneralization

Unlike random learner errors, overgeneralization errors are not random but systematic, representing a necessary stage in which learners reorganize fragmented linguistic knowledge into target-like grammatical competence (Brooks et al., 1999; Marcus et al., 1992; Pinker, 1989; King, 2006; Xiang et al., 2024). Their systematicity is shaped by multiple interacting factors, including cognitive development, structural properties, processing capacity, and learning mechanisms (see Ellis, 2008 for a comprehensive overview). These factors have been formalized in several theoretical frameworks. Overgeneralization has been documented across both monolingual and bilingual acquisition (Tamburelli, 2008) and can be systematically accounted for by four frameworks, which are Markedness Theory (Eckman, 1977, 2008; Gass and Selinker, 1983), U-shaped behavioral development (Kellerman, 1983, 1985; McLaughlin, 1990; Shirai, 1990; Marcus et al., 1992), Processability Theory (Pienemann, 1998, 2005), and form-meaning mapping (DeKeyser, 2000; DeKeyser, 2015; Ellis, 2006).

Markedness Theory (Eckman, 1977) provides a structural explanation for why certain linguistic forms are more susceptible to overgeneralization than others. Eckman’s (1977) Markedness Differential Hypothesis (MDH) predicts that learners are most likely to overgeneralize rules from unmarked structures to marked structures, particularly when a marked target structure corresponds to an unmarked L1 counterpart. In other words, overgeneralization is not uniform across linguistic features but is systematically constrained by markedness asymmetries. This MDH framework has been extended from phonology to syntax, where markedness has been shown to constrain overgeneralization patterns (Matsunaga, 2005; see also Archibald, 2021, for a review of markedness in phonology). Crucially, overgeneralization in SLA is often shaped by L1 transfer, as learners rely on existing L1 knowledge as a starting point (Kellerman, 1983; Odlin, 1989; Inagaki, 1997; Tamburelli, 2008). When L1 and L2 structures overlap, learners tend to transfer L1 patterns to L2 contexts, sometimes extending them beyond their legitimate scope. A case in point is Matsunaga’s (2005) study, which examined 29 Spanish-speaking and 28 Japanese-speaking learners of English using acceptability judgment and production tasks. The study treated the analytic causative (*make* + verb) as syntactically marked because it requires an additional syntactic layer beyond the lexical argument structure, unlike lexical causatives such as *break*. Results showed that Japanese learners at the lower proficiency level, whose L1 allows alternation with verbs like *disappear*, overgeneralized lexical causatives more than Spanish learners, extending the relatively unmarked pattern to contexts requiring the marked analytic causative. However, this cross-linguistic difference was attenuated among upper-proficiency learners (Matsunaga, 2005), suggesting that marked target structures are particularly vulnerable to rule-overextension driven by L1 transfer, though learners can recover from this transfer effect with increased proficiency. The study thus provides empirical evidence that structural markedness, in conjunction with L1 transfer, systematically shapes overgeneralization patterns in SLA.

While structural markedness and L1 transfer may predict where overgeneralization is likely to occur, whether this tendency materializes as errors depends on learners’ processing constraints (Pienemann, 1998, 2005). Overgeneralization can emerge only once learners have attained the developmental stage that affords the relevant syntactic-mapping operations. Pienemann’s (1998) Processability Theory provides a systematic account of this mechanism, specifying the hierarchical sequence in which grammatical operations become accessible to learners and, consequently, the developmental stages at which overgeneralization is most likely to occur. Within these developmental windows, learners may follow a non-linear path marked by U-shaped behavioral development (Kellerman, 1985; McLaughlin, 1990; Shirai, 1990; King, 2006).

The phenomenon of U-shaped development is intricately linked to overgeneralization patterns. Kellerman (1985) characterized U-shaped behavioral development as a pattern in which language learners’ performance is initially accurate, then deviates from the target norm, and finally becomes accurate again. Within this trajectory, overgeneralization serves as the core manifestation of the mid-stage dip, as learners actively apply newly abstracted grammatical rules to contexts where they do not yet know the exceptions apply. From a cognitive perspective, U-shaped development reflects the process of restructuring, wherein learners reorganize their internal representational framework, leading to temporary performance declines that are subsequently overcome as expertise increases (McLaughlin, 1990; Shirai, 1990). As King (2006) observed, the apparent “back-sliding” observed in children’s language development, where accuracy declines during the overgeneralization phase, is in fact an important sign of linguistic progress.

While U-shaped behavior in FLA and SLA appears similar in surface form, with accuracy rising, then falling, and then rising again, its underlying nature may differ fundamentally (Shirai, 1990). In L1 acquisition, U-shaped behavior is typically driven by rule overgeneralization: children abstract a rule (e.g., *-ed* for past tense) and apply it to exceptions, yielding forms like *goed, before later refining the system (Karmiloff-Smith, 1986). In SLA, however, the mid-stage “dip” may not reflect rule overgeneralization in the same sense. Instead, it often arises from shifting sensitivity to L1–L2 transferability, task demands, or instructional effects (Kellerman, 1985; Shirai, 1990). This raises the question of whether, and under what conditions, rule overgeneralization also plays a central role in L2 U-shaped development at the syntactic level, particularly in cases where learners face complex form-meaning mappings. These issues remain to be addressed in the present study, which focuses on the acquisition of Japanese psychomime verb constructions.

U-shaped development describes the trajectory of overgeneralization, but explaining how learners actually achieve the transition from erroneous overuse to target-like accuracy requires a more fine-grained account, one that addresses both the cognitive prerequisites (DeKeyser, 2000) and the instructional mechanisms (Ellis, 2006) underlying successful mapping refinement. DeKeyser (2000), testing Bley-Vroman’s (1988) Fundamental Difference Hypothesis, found that adult L2 success depends on verbal analytical ability, in that among adult arrivals, grammaticality judgment scores correlated with aptitude, but not among child arrivals, and all adult learners who scored within the child range possessed high aptitude, suggesting that analytical ability is a necessary condition for mapping refinement. This frames overgeneralization as a mapping problem. Learners establish overly broad form-meaning associations that require systematic refinement, with high-aptitude learners able to constrain overgeneralization through conscious analysis, while low-aptitude learners may remain fossilized. DeKeyser (2000) further identified perceptual salience as mediating this process, in that highly salient structures are acquired even by low-aptitude learners, whereas low-salience, abstract patterns (precisely those most vulnerable to overgeneralization) require high analytical ability for their resolution. Ellis (2006) provides an SLA perspective on how grammar can best be taught for implicit knowledge. He distinguishes between input-based and production-based instruction, and within the input-based option identifies interpretation tasks (Ellis, 1995) as a means of drawing learners’ attention to form-meaning mappings. The rationale for such tasks rests on a computational model of L2 acquisition, according to which acquisition takes place as learners comprehend and process input. By directing learners’ attention to the connections between grammatical forms and the meanings they realise, interpretation tasks facilitate the kind of form-meaning mapping that is foundational to the development of implicit knowledge. This pedagogical approach is underpinned by the weak interface position (Ellis, 1993), according to which explicit knowledge primes noticing and cognitive comparison (Schmidt, 1990). Together, DeKeyser (2000) and Ellis (2006) offer a two-tiered framework for understanding how learners overcome overgeneralization. At the cognitive level, the resolution of overgeneralization depends on analytical ability. High-aptitude learners can consciously refine overly broad form-meaning mappings, whereas low-aptitude learners may remain fossilized at the U-shaped dip. At the pedagogical level, instruction facilitates this refinement by providing explicit knowledge, promoting noticing, and offering corrective feedback, thereby enabling learners to progressively differentiate appropriate from inappropriate contexts of use.

Grounded in the theoretical perspectives outlined above, the present study examines how L2 learners acquire the syntactic properties of three types of Japanese psychomimes that differ in markedness and form-meaning mapping transparency. Markedness Theory predicts the differential vulnerability to overgeneralization across the three word classes and accounts for L1-transfer-induced rule extension. Processing constraints further determine the developmental stages at which such overgeneralization errors are most likely to emerge. The U-shaped developmental model captures the dynamic, non-linear fluctuations in acquisition performance over time. Form-meaning mapping theory illuminates the cognitive mechanism through which learners gradually refine syntactic-semantic associations and suppress erroneous overgeneralized rules. Collectively, these complementary perspectives establish an integrative theoretical framework for understanding the structural, processing, developmental, and form-meaning factors shaping overgeneralization.

### 2.5 The current study

This study investigates three categories of psychomime verbs distinguished by NP usage: mandatory NP attachment for physical NPs, NP suppression for psychological NPs, and optional NP attachment for both physical and psychological NPs. We conducted NP attachment judgement experiments with Japanese native speakers (JNSs) and Chinese-speaking learners of Japanese (CSLJs) to address the following research questions:

RQ1. Do JNSs apply conventional NP attachment rules to psychomimes? To what extent does semantic abstractness impact their NP attachment use?

RQ2. How do CSLJs construct syntactic structures for different psychomime types, and do they exhibit NP attachment overgeneralization? What influence does L2 grammatical proficiency have on such overgeneralization?

## 3 Methods

### 3.1 Experimental overview

Drawing on Akita’s (2010) classification, this study carried out two experiments. Experiment 1 used three categories of psychomime verbs (mandatory NP attachment, optional NP attachment and NP suppression) as the experimental materials. Seventy-six Japanese native university students completed the NP attachment judgement task to explore their application of attachment rules and verify the rationality of Akita’s three-class division against Kageyama’s (2007) two-class division.

In Experiment 2, 88 Chinese undergraduate Japanese majors participated in the identical judgement task. We investigated whether these L2 learners could master NP attachment rules like JNSs do. An L2 grammar test was also included to analyse how L2 grammatical proficiency influences learners’ use of NP attachment and suppression. All of the tasks were completed in the form of paper-and-pencil tests.

### 3.2 Design of the NP-attachment judgement experiment

The stimulus words for the NP attachment judgement experiment were selected with reference to the work of Connelly et al. (2016). In the preliminary exploratory experiment conducted by Connelly et al. (2016), a 5-point scale was employed to ask 7 JNSs to evaluate the naturalness of three classes of psychomimes with NP attachment. The results indicated that Class 3 did not exhibit NP suppression, in contrast to the theory proposed by Akita (2010). This inconsistency might be attributed to (1) the limited number of participants, which rendered the sample and data insufficiently extensive or representative, and (2) issues with the frequency of stimulus word usage and sentence naturalness, indicating a lack of rigor and accuracy in the experimental materials.

To address these methodological limitations and to provide a more robust test of the NP attachment patterns with a larger sample, the present study adopted an experimental design that systematically expanded upon the framework of Connelly et al. (2016). As shown in Table 1, in our test, 6 words were selected from each of the three categories of psychomimes, for a total of 18 words.

**Table 1:** Three categories of experimental stimulus words.

Types	ABAB-type psychomimes	Pronunciation	Mandarin counterpart	Presence of NP (Akita, 2010)	JLPT level	Mention in textbooks	Frequency in BCCWJ
Class 1	ずきずき	*/zuki-zuki/*	阵阵剧痛	Yes	Outside	Outside	96
	がんがん	*/gan-gan/*	嗡嗡胀痛	Yes	Outside	Outside	452
	しょぼしょぼ	*/syobo-syobo/*	眼睛酸涩	Yes	Outside	Outside	59
	きりきり	*/kiri-kiri/*	绞痛	Yes	Outside	Outside	105
	ちかちか	*/tika-tika/*	眼花	Yes	Outside	Outside	70
	ひりひり	*/hiri-hiri/*	灼痛感	Yes	Outside	Outside	138
Class 2	うきうき	*/uki-uki/*	喜气洋洋	Yes/None	Outside	Outside	216
	わくわく	*/waku-waku/*	兴奋期待	Yes/None	Outside	Outside	893
	くらくら	*/kura-kura/*	晕乎乎	Yes/None	Outside	Outside	188
	むかむか	*/muka-muka/*	恶心	Yes/None	Outside	Outside	111
	うずうず	*/uzu-uzu/*	跃跃欲试	Yes/None	Outside	Outside	119
	さばさば	*/saba-saba/*	爽快	Yes/None	Outside	Outside	68
Class 3	ぷりぷり	*/puri-puri/*	气鼓鼓	None	Outside	Outside	125
	うじうじ	*/uzi-uzi/*	犹豫不决	None	Outside	Outside	54
	ぐたぐた	*/guta-guta/*	瘫软无力	None	Outside	Outside	11
	かりかり	*/kari-kari/*	焦躁易怒	None	Outside	Outside	284
	くよくよ	*/kuyo-kuyo/*	耿耿于怀	None	Outside	Outside	195
	ぴりぴり	*/piri-piri/*	精神紧张	None	Outside	Outside	210

None of the 18 psychomimes are included in the Japanese Language Proficiency Test (JLPT).^1^ Stimuli for Class 1 and Class 2 were adopted from Connelly et al. (2016). For Class 3, two originally selected items from Connelly et al. (2016), *kosekose* and *magomago*, were replaced with *karikari* and *puripuri*, which were selected from the lexical inventory of Akita (2010). The replacement was made for two reasons. First, *kosekose* was reported as a rarely used word by JNSs in a prior study, and its low familiarity might have confounded participants’ judgments. Second, *magomago* falls within the JLPT N2–N3 vocabulary range, and its inclusion could have introduced bias due to differential lexical familiarity among L2 learners. By contrast, the two replacement items, *karikari* and *puripuri*, are off-level words that do not appear in any JLPT vocabulary lists. Additionally, none of the stimuli were covered in the intensive reading textbooks for the CSLJs, minimizing the likelihood that the participants had learned these words in university courses.

To control for word frequency effects, we retrieved the token frequencies of the 18 target psychomimes from the Balanced Corpus of Contemporary Written Japanese (BCCWJ). Although *gutaguta* occurred only 11 times and thus had a low frequency, it was retained as a stimulus item because Connelly et al. (2016) did not rule out its use. Additionally, a pre-experiment survey of five JNSs confirmed that no rare words appeared in the stimulus list. However, given that word frequency varied across the three psychomime categories, a *post hoc* Pearson correlation analysis was conducted between word frequency and NP attachment rates based on the data from the CSLJs (see Section 5). The results revealed a weak and non-significant correlation between word frequency and NP attachment rates across the 18 stimulus items, *r* (16) = 0.15, *ns*, with word frequency accounting for only about 2.25% of the variance in attachment rate (*r^2^* = 0.0225), which corresponds to a small effect size according to Cohen’s (1988) conventions, suggesting that frequency did not systematically bias the attachment judgments in the present dataset. To further examine whether word frequency influenced learners’ comprehension difficulty, we also conducted a *post hoc* Pearson correlation analysis between word frequency and distractor selection rates across the 18 items. A moderate negative correlation was observed, *r* (16) = −0.398, *ns, r^2^* = 0.1584, indicating that higher frequency items tended to yield slightly lower distractor rates, though this association did not reach statistical significance. The non-significant correlation suggests that comprehension difficulty, as indexed by distractor selection, did not systematically vary with word frequency in the present study. Neither correlation reached significance, suggesting that word frequency effects did not substantially confound the main findings.

The NP attachment judgement experiment adopted a single-choice format in which the participants selected the optimal answer from four options. The test comprised 18 items, with one question designed for each target psychomime (see Table 2 for detailed questions and options). The test design was adapted from Connelly et al. (2016), and all of the psychomimes were presented in the canonical ‘psychomime + *suru*’ verb form. To avoid confusion for L2 learners caused by the mixed use of Japanese and English personal names in the original stimuli, all names in the present test were standardized to the uniform ‘surname + *san*’ (‘Mr.’, ‘Mrs.’, ‘Miss’, or ‘Ms.’) format. Additionally, new test items were developed on the basis of dictionary examples for *karikari* and *puripuri*, which replaced the original stimuli *kosekose* and *magomago*. The test item for *uziuzi* was also revised, as participants in Connelly et al.’s (2016) study reported contextual mismatches for this item. Finally, five JNSs reviewed all 18 test questions in a pre-experiment evaluation, verifying the appropriateness of each item’s contextual compatibility.

**Table 2:** Questions, target options, and distractors on the psychomime test.

Types	Stimulus words	NPs	Questions and options			
Class 1	ずきずき *zukizuki*	Head	昨晩，田中さんは遅く寝たので，今日は朝から( )**ずきずき**している。			
			Last night, Tanaka slept late, so today his **head** has been feeling **a throbbing pain** since the morning.			
			A. 泡が bubble	B. 椅子が chair	C. 頭が head	D. 無 none
	がんがん *gangan*	Head	昨晩，山下さんはたくさんお酒を飲んで，今日は二日酔いで( )**がんがん**している。			
			Last night, Yamashita drank a lot, so today he has a hangover and his **head** is feeling **a pounding pain**.			
			A. 机が desk	B. 頭が head	C. 石が stone	D. 無 none
	しょぼしょぼ *syobosyobo*	Eyes	一日中，本を読んでいたら，鈴木さんは( )**しょぼしょぼ**してきた。			
			After reading books all day, Suzuki’s **eyes** started to become **bleary and sore**.			
			A. 目が eyes	B. 建物が buildings	C. 苗が seedlings	D. 無 none
	きりきり *kirikiri*	Stomach	松本さんは将来のことを考えて，不安で( )**きりきり**してきた。			
			Matsumoto is anxious when thinking about the future, and his **stomach** begins to feel **a sharp pain**.			
			A. 家が house	B. 胃が stomach	C. 車が car	D. 無 none
	ちかちか *tikatika*	Eyes	この工業地帯は大気汚染がひどいので，佐藤さんは( )**ちかちか**してきた。			
			Due to severe air pollution in this industrial area, Sato’s **eyes** began to **feel intermittent prickling**.			
			A. 目が eyes	B. 鉛筆が pencils	C. 鳥が birds	D. 無 none
	ひりひり *hirihiri*	Mouth	山田さんは辛いものを食べたので，( )**ひりひり**している。			
			Yamada ate something spicy, so his **mouth** is **tingling with a burning sensation**.			
			A. 口が mouth	B. 用紙が paper	C. 森が forest	D. 無 none
Class 2	うきうき *ukiuki*	Heart/none	明日から夏休みが始まると思うと，本田さんは( )**うきうき**する。			
			When Honda thinks about the summer vacation starting tomorrow, his **heart**/he feels **cheerful**.			
			A. 心が heart	B. 梅が plum	C. 米が rice	D. 無 none
	わくわく *wakuwaku*	Chest/none	明日，好きな人に会うと思うと，中村さんは( )**わくわく**する。			
			Thinking to meet the person he loves tomorrow, Nakamura’s **chest**/Nakamura is feeling **excited with anticipation**.			
			A. 窓が window	B. 村が village	C. 胸が chest	D. 無 none
	くらくら *kurakura*	Head/none	恐ろしい光景を見たので，山本さんは( )**くらくら**していた。			
			Having witnessed a terrifying scene, Yamamoto’s **head**/Yamamoto was feeling **dizzy**.			
			A. 草が grass	B. 地震が earthquake	C. 頭が head	D. 無 none
	むかむか *mukamuka*	Chest/none	森さんはお酒に弱いので，お酒を飲むと必ず( )**むかむか**する。			
			Mori is sensitive to alcohol, so his **chest**/he feels **nauseous** every time after drinking.			
			A. 空が sky	B. 村が village	C. 胸が chest	D. 無 none
	うずうず *uzuuzu*	Heart/none	小野さんは早くパーティーに行きたくて，( )**うずうず**している。			
			Ono is eager to go to the party, his **heart**/he is feeling **restless with excitement**.			
			A. 紅茶が black tea	B. 心が heart	C. 塩が salt	D. 無 none
	さばさば *sabasaba*	Mood/none	長年の悩みが解消され，藤本さんは( )**さばさば**している。			
			After resolving a long-standing worry, Fujimoto’s **mood**/Fujimoto is feeling **refreshed and unburdened.**			
			A. 金属が metal	B. 辞書が dictionary	C. 気持ちが mood	D. 無 none
Class 3	ぷりぷり *puripuri*	None	櫻井さんは友達を3時間待って，( )**ぷりぷり**している。			
			Sakurai has been waiting for a friend for three hours, and he/his **heart** is **fuming with anger**.			
			A. 心が heart	B. 本が book	C. 海が sea	D. 無 none
	うじうじ *uziuzi*	None	土田さんは就職の面接がうまくいかず，( )**うじうじし**ている。			
			Tsuchida’s job interview did not go well, and he/his **heart** is **restless**.			
			A. 花が flower	B. 心が heart	C. 光が light	D. 無 none
	ぐたぐた *gutaguta*	None	田村さんは昨日運動し過ぎたので，今日は( )**ぐたぐた**している。			
			Tamura exercised too much yesterday, so today he/his **body** is **exhausted**.			
			A. 体が body	B. 岩が rock	C. 湖が lake	D. 無 none
	かりかり *karikari*	None	小林さんは夫が口うるさいので，つまらないことに( )**かりかり**する。			
			Because Kobayashi’s husband is nagging, so she/her **heart** is easily **distracted** by trivial matters.			
			A. 服が clothes	B. 心が heart	C. 道が road	D. 無 none
	くよくよ *kuyokuyo*	None	試験に失敗したので，水野さんは( )**くよくよ**している。			
			Because Mizuno failed an exam, she/her **heart** is **mopey**.			
			A. 塩が salt	B. 靴が shoes	C. 心が heart	D. 無 none
	ぴりぴり *piripiri*	None	入学試験を前に学生は( )**ぴりぴり**している。			
			Before the entrance exam, students/students’ **hearts** are **nervous**.			
			A. 魚が fish	B. 心が hearts	C. 写真が photos	D. 無 none

All of the words in the questions and options were basic vocabulary. We verified their difficulty with 15 beginner Japanese learners (average 11 months of learning), who translated every term correctly. Since 84 of the 88 participants were second-year Japanese majors, basic vocabulary did not interfere with the test results.

The four options consisted of 2 targets and 2 distractors. As shown in Question (1), one target option was the NP that matched the psychomime in the question, and the other was ‘*nasi*’, meaning that it did not require any additional NP. The remaining 2 options were distractors that were not related to the question sentences. If the CSLJs chose a distractor, then the participant did not fully understand the context in which the stimulus word appeared, and the data related to that part were excluded from the data analysis.

For example, in Question (1), the word *zukizuki* (‘throbbing pain’) belonging to Class 1 must be paired with the NP *atama-ga* (‘head’) to complete the sentence. Therefore, during the data analysis, only the data for the participants who selected the target options *atama-ga* and *nasi* were analysed to determine whether there was a significant difference. The data for the participants who selected the distractors *awa-ga* (‘bubble’) and *isu-ga* (‘chair’) were not included in the analysis.

Question (1):

昨晩，田中さんは遅く寝たので，今日は朝から(　　)**ずきずき**している。

A. awa-*ga* (‘bubble’) B. *isu-ga* (‘chair’) ☑C. *atama-ga* (‘head’) D. *nasi* (‘none’)

To examine the internal consistency reliability of the experimental materials, a Cronbach’s alpha analysis was conducted on all 18 items. The results showed that the overall reliability coefficient was *α* = 0.76 (*N* = 88), indicating acceptable internal consistency reliability (Nunnally, 1978).

### 3.3 L2 grammar test design and L2 grammatical proficiency grouping for CSLJs

Previous research has established that proficiency effects significantly influence L2 learners’ overgeneralization patterns. Taylor (1975) observed that as proficiency increases, learners tend to produce more overgeneralizations of L2 rules, suggesting a developmental shift from reliance on L1 transfer to the active application of L2-based generalizations. Matsunaga (2005) further found that while advanced learners can partially recover from such errors, the persistence of certain non-target forms indicates that argument structure knowledge is acquired gradually, with higher proficiency associated with more target-like performance. More recently, Wei et al. (2026) demonstrated that higher-proficiency Chinese EFL learners showed stronger sensitivity to entrenchment, preemption, and semantic constraints, suggesting that increased proficiency enhances learners’ ability to use statistical and structural information to constrain overgeneralization. These findings suggest that proficiency modulates overgeneralization in a complex manner: it strengthens L2-based generalization and learners’ sensitivity to probabilistic constraints, while higher proficiency levels are associated with more refined, target-constrained use of argument structure patterns.

To whether the proficiency effects documented in previous research also apply to the acquisition of Japanese psychomime constructions, the present study included a measure of L2 grammatical knowledge in addition to the psychomime test. The L2 grammar test adopted in this study was developed by Hayakawa and Tamaoka (2015) and contained 36 single-choice questions. The test is organized around three structural categories: morphological inflections, local dependency, and complex structure. Each category contains 12 questions, yielding a total of 36 items. Hayakawa and Tamaoka (2015) administered this test to 143 CSLJs at a university in eastern China, and the Cronbach’s alpha reliability coefficient was high (*α* = 0.82). This test has thus been validated with CSLJs and provides a stable measure of syntactic knowledge, making it suitable for assessing L2 grammatical proficiency in the present study. In the present sample of 88 CSLJ participants, the Cronbach’s alpha reliability coefficient was *α* = 0.80, indicating good reliability and allowing for the accurate quantification of the participants’ L2 grammar competence.

The L2 grammar test had a total score of 36 points. The participants’ scores ranged from 9 to 34 points (M = 20.16, SD = 5.84). On the basis of the mean score and score distribution (for similar approaches, see Matsunaga, 2005), the participants were divided into three groups. 33 participants scoring 18–23 points were categorized into the middle-proficiency group (M = 20.82, SD = 2.02, close to the overall mean), 30 participants scoring 9–17 points were categorized into the low-proficiency group (M = 13.73, SD = 2.80), and 25 participants scoring 24–34 points were categorized into the high-proficiency group (M = 27.00, SD = 2.75).

A one-way ANOVA was performed to compare the mean scores across the three groups. A significant main effect of grammatical proficiency was observed, *F* (2, 85) = 190.43, *p* < 0.001, *ω*^2^ = 0.812 [0.735, 0.853]. According to Cohen’s (1988) benchmarks, this represents a large effect.

*Post hoc* multiple comparisons revealed significant differences between all of the groups, with the performance ordered as high-level > middle-level > low-level. These findings confirm that the three-tier grouping based on the performance of the grammar test is valid. Therefore, the categorization of the CSLJ participants into high-, middle-, and low-level grammatical proficiency groups on the basis of the L2 grammar test results was valid.

## 4 Experiment 1: NP-attachment judgement experiment for JNS participants

### 4.1 Participants

Experiment 1 was conducted at a university located in the Tokai region of Japan. A total of 76 Japanese university undergraduates (61 males, 15 females) whose native language was Japanese participated in the NP-attachment judgement experiment. The youngest participant was 17 years and 5 months old, and the oldest participant was 22 years and 9 months old (M = 19 years and 11 months, SD = 12 months).

### 4.2 Data analysis and results

#### 4.2.1 Chi-square goodness-of-fit test and one-sample *t* test for within-group NP attachment and suppression

We conducted a goodness-of-fit test using the chi-square distribution to examine whether the numbers of NP attachment (NP+) and NP suppression (NP−) responses differed significantly from chance (50%) for each of the 18 psychomime verbs. The results of the analysis are presented in Table 3. To control for family-wise error rate, Bonferroni correction was applied, with the significance level adjusted to *α* = 0.05/18 = 0.0028.

**Table 3:** Difference analysis between JNS NP attachment and NP suppression.

Types	Stimulus words	NP presence	NP+ number	NP− number	NP+ rate	*χ^2^* (1)	*p*	NP preference
Class 1	*tikatika*	Yes	73	3	96.05%	Ceiling effect		NP+
	*kirikiri*	Yes	76	0	100.00%			NP+
	*hirihiri*	Yes	76	0	100.00%			NP+
	*gangan*	Yes	76	0	100.00%			NP+
	*syobosyobo*	Yes	76	0	100.00%			NP+
	*zukizuki*	Yes	76	0	100.00%			NP+
Class 2	*mukamuka*	Yes/None	69	7	90.79%	50.58	<0.001***	NP+
	*kurakura*	Yes/None	69	7	90.79%	50.58	<0.001***	NP+
	*ukiuki*	Yes/None	60	16	78.95%	25.47	<0.001***	NP+
	*wakuwaku*	Yes/None	58	18	76.32%	21.05	<0.001***	NP+
	*sabasaba*	Yes/None	58	18	76.32%	21.05	<0.001***	NP+
	*uzuuzu*	Yes/None	30	46	39.47%	3.37	*ns*	—
Class 3	*karikari*	None	10	66	13.16%	41.26	<0.001***	NP−
	*kuyokuyo*	None	10	66	13.16%	41.26	<0.001***	NP−
	*puripuri*	None	11	65	14.47%	38.37	<0.001***	NP−
	*uziuzi*	None	24	52	31.58%	10.32	=0.001***	NP−
	*gutaguta*	None	35	41	46.05%	0.47	*ns*	—
	*piripiri*	None	36	40	47.37%	0.21	*ns*	—

*N* = 76. “NP Presence” = whether the NP is grammatically required [the significant response direction after Bonferroni correction (*p* < 0.0028)]. “NP+” = NP attachment choice, “NP−” = NP suppression choice, “—” indicates no significant preference. *ns*, not significant (*p* ≥ 0.05 uncorrected). ***p < 0.001. Light shading indicates no significant difference after Bonferroni correction.

Class 1 showed an NP attachment rate of nearly 100% (M = 99.34%, SD = 1.61%), indicating a ceiling effect and confirming the strong preference of native speakers for NP attachment, which is consistent with the findings of Akita (2010).

In Class 2, five items (*mukamuka*, *kurakura*, *ukiuki*, *wakuwaku*, and *sabasaba*) showed significantly more NP attachments than suppressions, with NP attachment rates ranging from 76.32 to 90.79%. No significant difference was observed for *uzuuzu*. A one-sample *t* test indicated that the overall NP attachment rate of Class 2 (M = 75.44%, SD = 18.86%) was significantly above the 50% chance level, *t*(5) = 3.30, *p* < 0.05, *g* = 1.13 [0.15, 2.06].

For the six Class 3 items, *karikari*, *kuyokuyo*, *puripuri*, and *uziuzi* showed significantly more NP suppressions than attachments. No significant difference was found for *gutaguta* or *piripiri*. A one-sample *t* test further revealed that Class 3’s overall NP suppression rate (M = 72.37%, SD = 16.35%) was significantly above the 50% chance level, *t*(5) = 3.35, *p* < 0.05, *g* = 1.15 [0.16, 2.09], indicating a distinct NP suppression tendency among native speakers for all six Class 3 items.

The results reveal a general tendency for Japanese native speakers to use NP attachment with Class 2 items. However, this finding alone cannot disprove Akita’s (2010) classification. To examine lexical differences across the three categories, a one-way repeated-measures ANOVA was conducted on their mean NP attachment rates, as reported below.

#### 4.2.2 One-way repeated-measures ANOVA for NP attachment across groups

Prior to the analysis, the data were coded on the basis of the participants’ responses: NP attachment was assigned a score of 1, and NP suppression was assigned a score of 0. Each psychomime category had a maximum total score of 6. As presented in Table 4, Figure 2, the main effect of psychomime type was significant [*F* (1.70, 127.74) = 253.55, *p* < 0.001, *ω_G_*^2^ = 0.66]. According to the criteria of Field (2013), the effect size (generalized omega-squared) was large.

**Table 4:** Results of a one-way repeated-measures ANOVA on NP attachment scores.

Types	M (NP+)	SD (NP+)
Class 1	5.96	0.20
Class 2	4.53	1.36
Class 3	1.66	1.65
ANOVA results: *F* (1.70, 127.74) = 253.55, *p* < 0.001, *ω_G_*^2^ = 0.66		
Class 1 vs. Class 2	*t* (75) = 9.20, *p* < 0.001, *g* = 1.47 [1.11, 1.83]	
Class 2 vs. Class 3	*t* (75) = 12.62, *p* < 0.001, *g* = 1.89 [1.51, 2.27]	
Class 1 vs. Class 3	*t* (75) = 22.20, *p* < 0.001, *g* = 3.65 [3.13, 4.18]	
Scheffé’s multiple comparison test results: Class 1 > Class 2 ≠ Class 3		

**Figure 2:**
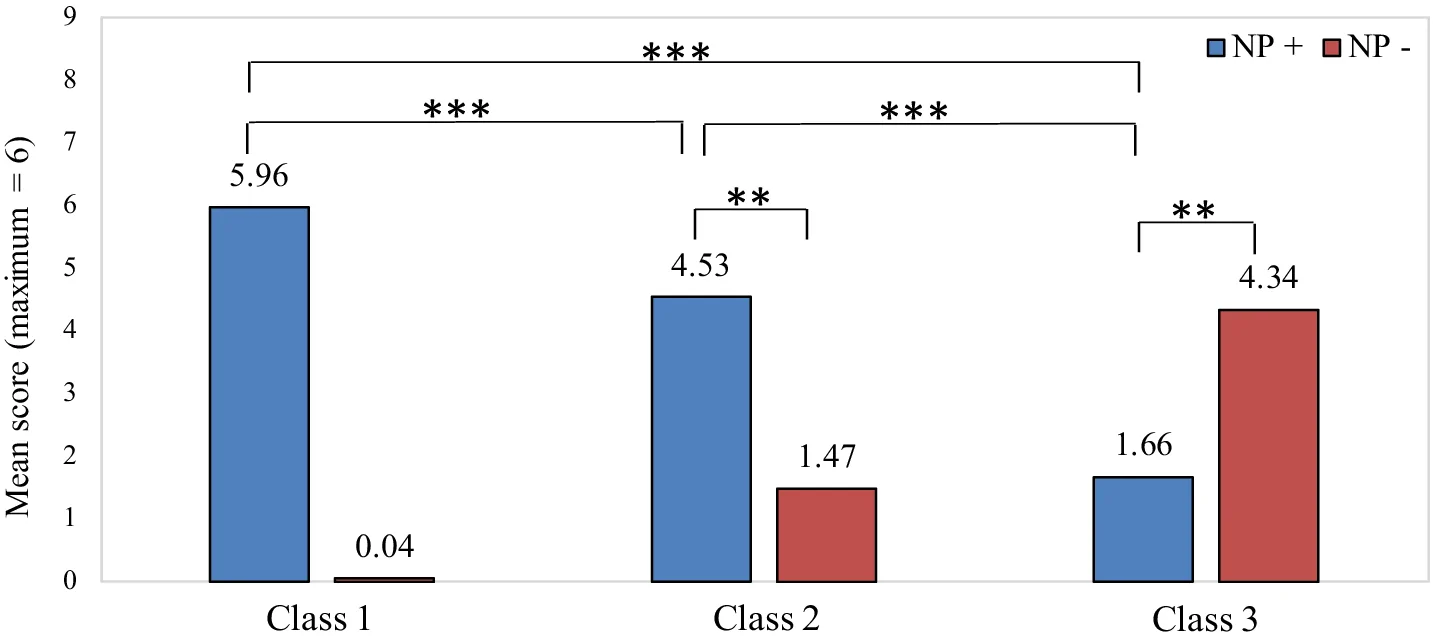
Intergroup and within-group significance among the three psychomime types.

*Post-hoc* multiple comparisons using Scheffé’s test revealed significant differences across all three groups. The mean NP attachment scores followed the order of Class 3 (M = 1.66, SD = 1.65) < Class 2 (M = 4.53, SD = 1.36) < Class 1 (M = 5.96, SD = 0.20). Specifically, compared with Class 2, Class 1 yielded significantly higher scores, *t* (75) = 9.20, *p* < 0.001, *g* = 1.47 [1.11, 1.83]. These findings indicate that Class 1 and Class 2 belong to distinct lexical categories, whereas Class 3 is distinguished by a predominant tendency for NP suppression. Collectively, the three classes represent psychomime verbs with different lexical conceptual structures (Class 1 > Class 2 ≠ Class 3).

The analytical results of Experiment 1 fully confirm that Akita’s (2010) three-category classification theory is overall more valid than Kageyama’s (2007) two-category counterpart is.

## 5 Experiment 2: NP-attachment judgement experiment for CSLJ participants

In Experiment 2, all of the 54 single-choice items were presented randomly, including 18 questions on psychomime NP attachment and 36 L2 grammar questions, each with four response options.

### 5.1 Participants

Experiment 2 was administered at a university in eastern China. The 88 participants were Chinese undergraduate students majoring in Japanese (21 males, 67 females): 4 sophomores, 37 juniors and 47 seniors, none of whom had studied in Japan. Their ages ranged from 19 years and 1 month to 23 years and 4 months (M = 23 years and 4 months, SD = 11 months), with a mean age comparable to that of the native Japanese participants in Experiment 1.

### 5.2 Data analysis and results

#### 5.2.1 Chi-square goodness-of-fit test and one-sample *t* test on NP attachment and NP suppression within the group

Prior to the main analyses, we examined the proportion of distractor responses across all items. A total of 116 out of 1,584 responses (7.32%) were excluded as distractors, with item-level rates ranging from 0% (*wakuwaku*) to 13.64% (*karikari* and *ukiuki*). The overall low distractor rate suggests that comprehension difficulty was minimal and did not substantially affect the main results. These trials were therefore excluded from subsequent analyses of NP attachment preferences.

We adopted chi-square goodness-of-fit tests and one-sample *t* tests for within-group comparisons of NP attachment (NP+) and NP suppression (NP−). We first performed a chi-square goodness-of-fit test on the counts of these two response types, with the results shown in Table 5.

**Table 5:** Difference analysis between NP attachment and NP suppression among the CSLJs.

Types	Stimulus words	NP presence	NP+ number	NP− number	NP+ rate	*χ^2^*(1)	*p*	NP preference
Class 1	*gangan*	Yes	75	8	90.36%	54.08	<0.001***	NP+
	*hirihiri*	Yes	67	11	85.90%	40.21	<0.001***	NP+
	*zukizuki*	Yes	66	16	80.49%	30.49	<0.001***	NP+
	*syobosyobo*	Yes	65	16	80.25%	29.64	<0.001***	NP+
	*tikatika*	Yes	50	31	61.73%	4.46	<0.05*	*—*
	*kirikiri*	Yes	36	46	43.90%	1.22	*ns*	*—*
Class 2	*sabasaba*	Yes/None	44	36	55.00%	0.80	*ns*	*—*
	*mukamuka*	Yes/None	47	37	55.95%	1.19	*ns*	*—*
	*ukiuki*	Yes/None	39	37	51.32%	0.05	*ns*	*—*
	*uzuuzu*	Yes/None	39	39	50.00%	0	*ns*	*—*
	*kurakura*	Yes/None	29	52	35.80%	6.53	<0.05*	*—*
	*wakuwaku*	Yes/None	61	27	69.32%	13.14	<0.001***	NP+
Class 3	*piripiri*	None	43	40	51.81%	1.01	*ns*	*—*
	*karikari*	None	39	37	51.32%	0.05	*ns*	*—*
	*puripuri*	None	40	45	47.06%	0.29	*ns*	*—*
	*kuyokuyo*	None	42	42	50.00%	0	*ns*	*—*
	*gutaguta*	None	57	25	69.51%	12.49	<0.001***	NP+
	*uziuzi*	None	58	26	69.05%	12.19	<0.001***	NP+

*N* = 88. “NP Presence” = whether the NP is grammatically required [the significant response direction after Bonferroni correction (*p* < 0.0028)]. “NP+” = NP attachment choice, “NP−” = NP suppression choice, “—” indicates no significant preference. ns, not significant (*p* ≥ 0.05 uncorrected). *p < 0.05, ***p < 0.001. Light shading indicates no significant difference after Bonferroni correction.

For the six Class 1 psychomimes, NP attachment was chosen significantly more often than suppression for *gangan, hirihiri, zukizuki, syobosyobo* and *tikatika*, whereas no significant difference emerged for *kirikiri*. A one-sample *t* test revealed that the mean NP attachment rate of Class 1 (M = 73.77%, SD = 17.59%) was significantly above the 50% chance level, *t*(5) = 3.31, *p* < 0.05, *g* = 1.14 [0.15, 2.07], suggesting that Chinese learners tend to use NP attachment to interpret Class 1 expressions.

Among the six Class 2 items, the attachment and suppression counts did not differ significantly for *sabasaba, mukamuka, ukiuki* and *uzuuzu*. *Wakuwaku* yielded more attachment choices, whereas *kurakura* received more suppression choices. Overall, the mean attachment rate of Class 2 (M = 52.90%, SD = 10.83%) did not differ significantly from 50%, *t*(5) = 0.66, *ns*, *g* = 0.23 [0.47, 0.90].

In Class 3, *gutaguta* and *uziuzi* had significantly more NP attachments than they did suppressions. Neither response type differed significantly for *piripiri, karikari, puripuri* or *kuyokuyo*. The overall mean NP attachment rate for Class 3 (M = 56.46%, SD = 10.07%) did not differ significantly from the 50% chance level, *t*(5) = 1.57, *ns*, *g* = 0.54 [0.23, 1.27]. These findings reveal an overgeneralization of NP attachment: Chinese learners frequently add NPs even to psychomimes that do not require such syntactic elements.

A Bonferroni-corrected significance level of *α* = 0.0028 (0.05/18) was applied to control for family-wise error rate. Items that were marginally significant at the uncorrected level (*tikatika* and *kurakura*, both *p* < 0.05) did not survive the correction and should therefore be interpreted with caution. At the category level, Class 1 items predominantly favored NP+ attachment (four out of six items remained significant after correction), while Class 2 and Class 3 each showed only one and two significant items, respectively. These findings indicate that CSLJs generally conform to NP attachment rules when processing Class 1 and Class 2 psychomimes, but exhibit a clear overgeneralization pattern in Class 3, mechanically extending the syntactic rules of Class 2 to Class 3 without distinguishing the lexical differences between them.

#### 5.2.2 Multiple linear regression analysis of predictors of L2 grammatical proficiency

To further examine the factors underlying this overgeneralization pattern and to assess the relative contribution of Class 1 and Class 3 performance to learners’ overall grammar proficiency, we conducted a multiple linear regression analysis. Class 1 accuracy rate was defined as L2 learners’ performance on the NP attachment condition (NP+), and Class 3 accuracy rate was defined as their performance on the NP suppression condition (NP−). This analysis aimed to determine whether the ability to correctly process these two conditions differentially predicted grammar proficiency, thereby providing insights into the cognitive mechanisms driving the observed overgeneralization in Class 3. Regression coefficient estimates are presented in Table 6.

**Table 6:** Multiple linear regression analysis predicting L2 grammar proficiency.

Predictor	*B*	*SE*	*β*	*t*	*p*	95% CI for *B*	VIF
(Constant)	11.98	2.34	—	5.11	<0.001***	[7.32, 16.64]	—
Class 1	1.33	0.41	0.33	3.21	<0.01**	[0.51, 2.15]	1.06
Class 3	0.98	0.42	0.25	2.36	<0.05*	[0.16, 1.81]	1.06

*N* = 88. *R*^2^ = 0.133, Adjusted *R*^2^ = 0.113. *p < 0.05, **p < 0.01, ***p < 0.001.

The overall regression model was statistically significant, *F* (2, 85) = 6.51, *p* < 0.01, *R^2^* = 0.13, adjusted *R^2^* = 0.11. Collinearity diagnostics confirmed that multicollinearity was not a concern, with Variance Inflation Factors (VIF) both at 1.06, well below the conventional threshold of 10. A visual inspection of the scatterplot of standardized residuals against standardized predicted values (see Figure 3) revealed no systematic pattern, supporting the independence assumption.

**Figure 3:**
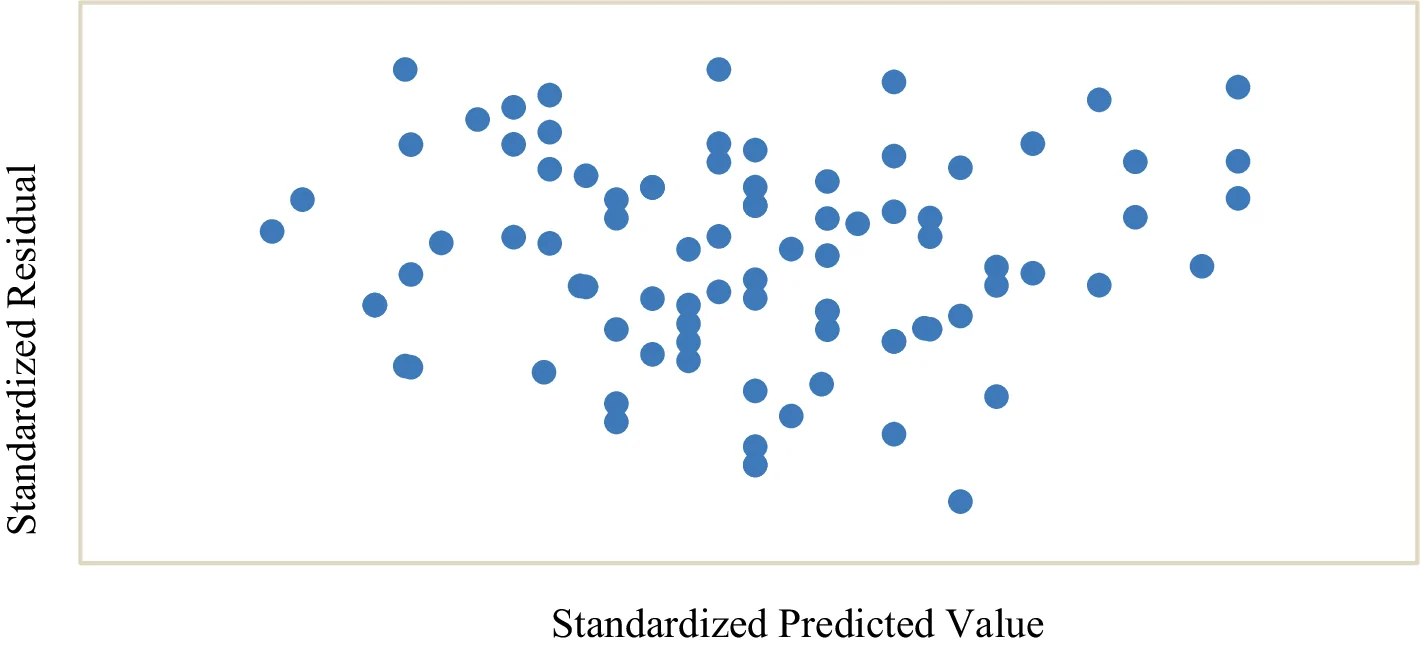
Scatterplot of standardized residuals against standardized predicted values.

Class 1 score emerged as a significant positive predictor of grammar proficiency (*β* = 0.33, *p* < 0.01), indicating that a one-unit increase in Class 1 score was associated with a 1.33-point increase in grammar proficiency, holding Class 3 constant. Similarly, Class 3 also significantly and positively predicted grammar proficiency (*β* = 0.25, *p* < 0.05), after controlling for class 1 score. When comparing the standardized coefficients (*β*), Class 1 score demonstrated a slightly stronger predictive power than Class 3. These findings suggest that both variables are independent and significant contributors to grammar proficiency, though a substantial portion of variance remains to be explained by other factors.

A supplementary analysis additionally entering Class 2 as a predictor revealed no significant independent effect (*B* = 0.33, SE = 0.40, *β* = 0.11, *t* = 0.84, *p* = 0.40), and the model fit did not improve (adjusted *R^2^* = 0.109) compared with the two-predictor model (adjusted *R^2^* = 0.113). Therefore, Class 2 was excluded from the final model.

#### 5.2.3 Factors predicting NP attachment: classification tree and logistic regression analyses

To complement the preceding multiple linear regression analysis, a classification tree analysis based on Classification and Regression Tree (CART) algorithm was further conducted to explore the factors predicting learners’ choice between NP attachment (NP+) and NP suppression (NP−). Unlike the regression analysis, which treated Class 1 and Class 3 accuracy rates as continuous predictors of grammar proficiency, the classification tree analysis treated learners’ response choice (NP+ vs. NP−) as the binary outcome variable. This approach allowed for the identification of optimal cutoff points in predictors that best distinguish between the two choice groups, revealing hierarchical relationships and interaction effects among variables (Breiman et al., 1984; Stewart and Stamm, 1991). Participants who selected neither option were excluded from this analysis. For model specification, response choice was set as the dependent variable, with grammatical proficiency, Class 1 choice rate, and Class 3 choice rate entered as predictors. The tree was grown using the Gini impurity criterion, and recursive partitioning was used to screen for the most discriminative predictors and their critical threshold values. Chi-square tests were applied to assess the significance of node splits.

The classification tree analysis (see Figure 4) revealed that psychomime type was the dominant factor affecting learners’ NP attachment choices [*χ^2^*(1) = 45.77, *p* < 0.001]. Class 1 (Node 1) formed a distinct cluster, whereas Classes 2 and 3 (Node 2) exhibited no significant differences and were grouped together. The overall NP attachment rate was significantly higher for Class 1 (M = 73.72%) than it was for the combined Classes 2 and 3 (M = 55.46%). Within Class 1, the attachment rate (M = 73.72%) greatly exceeded the suppression rate (M = 26.28%). This result suggests that accompanying NPs serve as core semantic elements for the CSLJs to interpret sentences with Class 1 psychomimes.

**Figure 4:**
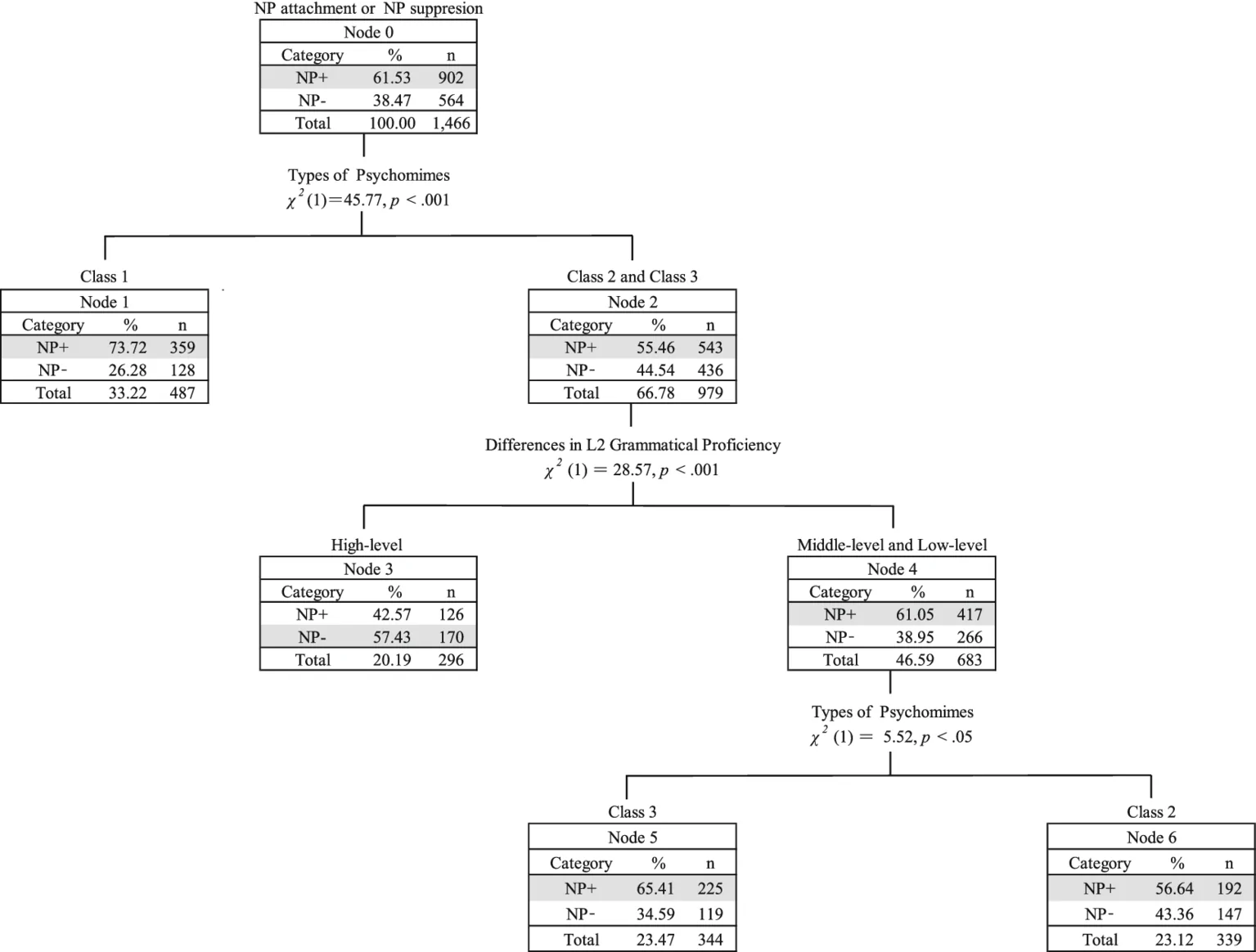
Results of the classification tree analysis.

With respect to Class 1, L2 grammatical proficiency had no significant effect on NP attachment. Specifically, no significant differences in the frequency of NP use were found among the high-, middle- and low-level groups. Collectively, these results indicate that, similar to the JNSs, all of the Chinese L2 Japanese learners in Experiment 2 rely on NP attachment to fully comprehend sentences containing Class 1 psychomimes.

With respect to Classes 2 and 3, the CSLJs did not use NPs as obligatorily as they did for Class 1. Even so, the overall NP attachment rate (M = 55.46%) remained higher than the suppression rate (M = 44.54%). For Class 3 in particular, NP suppression is the grammatically correct choice, yet the results revealed clear overgeneralization of NP attachment among the CSLJs. This finding indicates that the CSLJs processed Class 3 psychomime sentences in much the same way as they did Class 2: they relied on NP attachment to interpret the intended meaning.

In contrast to Class 1, L2 grammatical proficiency emerged as a significant predictor for Classes 2 and 3 [*χ^2^* (1) = 28.57, *p* < 0.001]. Further group comparisons revealed no significant difference in NP attachment rates between the middle- and low-proficiency groups. Notably, compared with those in the high-proficiency group, learners at these two levels were more inclined to use NP attachment. Compared with the attachment group (M = 42.57%), the high-proficiency group (Node 3) presented a significantly greater NP suppression rate (M = 57.43%). In contrast, the middle- and low-proficiency groups (Node 4) showed the opposite pattern: their NP attachment rate (M = 61.05%) was considerably higher than their suppression rate (M = 38.95%). Overall, these results indicate that for Class 2 and 3 psychomimes, middle- and low-proficiency learners preferred NP attachment, whereas high-proficiency learners tended to select NP suppression.

Furthermore, the psychomime type differed significantly between the middle- and low-proficiency groups [*χ^2^* (1) = 5.52, *p* < 0.05]. The probability of NP attachment in Class 3 (M = 65.41%, Node 5) was significantly greater than was that in Class 2 (M = 56.64%, Node 6). These results suggest that middle- and low-proficiency learners tended to use NP attachment more often for Class 3 than they do for Class 2. Specifically, they exhibited obvious overgeneralization of NP attachment rules to Class 3. When processing Class 3 items, high-proficiency learners correctly identified the nonobligatory nature of NPs, whereas less proficient learners failed to do so.

To further validate the robustness of the classification tree findings and to address the concern that proficiency was treated as a categorical variable, a binary logistic regression analysis was conducted. The dependent variable was learners’ overall NP attachment preference, coded as 1 for NP+ preference and 0 for NP− preference, derived from the total number of NP+ choices across all 18 items. Predictors included continuous proficiency scores and the frequency counts of NP+ choices for Class 1, Class 2, and Class 3 items (each ranging from 0 to 6). The results are presented in Table 7. The overall model was significant, *χ*^2^(4) = 81.05, *p* < 0.001, Nagelkerke *R^2^* = 0.81, with an overall classification accuracy of 97.70%. The results showed that Class 1 (*B* = 0.96, *p* < 0.05), Class 2 (*B* = 1.57, *p* < 0.001), and Class 3 (*B* = 1.31, *p* < 0.01) all significantly predicted the likelihood of choosing NP+, with Class 2 exhibiting the strongest effect. This suggests that learners’ performance on Class 2 items, which involve the integration of both physical and psychological dimensions of meaning, is the most sensitive indicator of their overall NP attachment preference. In contrast, proficiency scores did not reach significance (*B* = −0.05, *ns*). These results are consistent with the classification tree findings and suggest that the relationship between proficiency and NP attachment preferences may be non-linear, characterized by a critical threshold rather than a simple linear progression.

**Table 7:** Binary logistic regression results predicting NP+ preference.

Predictor	*B*	*SE*	Wald	*p*	Exp (*B*)	95% CI for Exp (*B*)
Constant	−10.17	3.05	11.09	<0.001***	0	—
Proficiency	−0.05	0.07	0.46	0.48	0.95	[0.82, 1.10]
Class 1	0.96	0.44	4.64	<0.05*	2.60	[1.09, 6.20]
Class 2	1.57	0.41	15.00	<0.001***	4.82	[2.18, 10.69]
Class 3	1.31	0.43	9.29	<0.01**	3.70	[1.59, 8.59]

*N* = 88. Nagelkerke *R*^2^ = 0.81. *p < 0.05, **p < 0.01, ***p < 0.001.

## 6 Discussion

### 6.1 Experiment 1

In the NP-attachment judgement experiment for JNSs, significant differences were observed in the NP attachment of three types of psychomime verbs, specifically demonstrating that ‘Class 1 > Class 2 ≠ Class 3’. Therefore, Akita’s (2010) three-category classification is more reasonable than Kageyama’s (2007) two-category classification is. Furthermore, Akita’s (2010) NP-attachment rules for the three types of psychomime verbs are more applicable to FLA.

The NP attachment rate for Class 1 was close to 100% (M = 99.34%). However, there were exceptions in the experimental stimulus of Classes 2 and 3 that did not conform to the NP attachment rules because of the varying semantic intensity and familiarity of polysemous psychomime meanings in actual usage.

The six Class 2 psychomimes were split into three subgroups by attachment rates: *mukamuka* and *kurakura* (M = 90.79%); *ukiuki*, *wakuwaku* and *sabasaba* (M = 77.20%); and the standalone item *uzuuzu* (M = 39.47%). As the semantic focus moves from concrete physical sensations to abstract psychological states, NP attachment becomes less acceptable.

Class 3 psychomimes yielded an NP suppression rate of M = 72.37%. Although not absolute, sentences without NPs sounded much more natural. No significant difference between attachment and suppression was observed for *gutaguta* and *piripiri*, two words that describe both physical feelings and mental states with blurred semantic boundaries. For the remaining four words, the participants strongly preferred NP suppression. Dictionary entries indicate that *gutaguta* and *piripiri* function dually as sensory verbs for physical experiences and as stative verbs for abstract emotions, with little practical distinction between these two senses in daily use.

Collectively, the results demonstrate a rule: psychomime verbs with concrete perceptual meanings tend to co-occur with NPs, whereas those carrying abstract or emotional meanings are less likely to take accompanying NPs. This finding explains why certain polysemous target words in the present study did not comply fully with Akita’s (2010) NP attachment rules. Future research could benefit from applying the methodologies utilized in this study to validate systematically the remaining psychomimes outlined in Akita’s (2010) classification.

Similarly, we may derive the insight that linguistic classification theories cannot remain at the level of pure theoretical speculation. With experimental paradigms including judgement tasks, researchers can assess quantitatively the soundness and validity of such classifications and support theoretical assumptions with objective data.

### 6.2 Experiment 2

#### 6.2.1 Category-specific overgeneralization in NP attachment

The results from the NP attachment experiment involving 88 CSLJs show that L2 learners struggle more with Class 3 psychomimes, which require NP suppression, than with Class 1 and Class 2 psychomimes, which allow NP attachment.

For Class 1, the NP attachment rate (M = 73.77%) was well above the 50% chance level. In contrast, the attachment rates for Classes 2 (M = 52.90%) and 3 (M = 56.46%) were close to the 50% chance level. CSLJs treated Classes 2 and 3 as a unified category and failed to follow the NP suppression rule in Class 3. This pattern suggests that the acquisition of NP attachment rules is category-specific, with more semantically abstract psychomime categories posing greater challenges for L2 learners. This finding aligns with Markedness Theory (Eckman, 1977): learners overgeneralize rules from unmarked structures (Class 2) to marked structures (Class 3), particularly when a marked target structure corresponds to an unmarked L1 counterpart.

However, considerable individual variation was observed. A subsequent logistic regression analysis revealed that this variation was systematically related to learners’ performance on Class 1, Class 2, and Class 3 items, with higher NP+ scores on these categories significantly predicting NP+ preference. This indicates that learners’ performance on these categories was not random but systematically related to their overall attachment preference. Class 2 emerged as the strongest predictor. With an Exp(*B*) of 4.82, substantially higher than that of Class 1 (2.60) and Class 3 (3.70), this finding suggests that learners’ performance on Class 2 items is the most sensitive indicator of their overall NP attachment preference. This result is theoretically meaningful when viewed through Akita’s (2010) classification, which positions Class 2 (somatopsych-mimetics) at an intermediate point on a continuum from concrete bodily sensation (Class 1) to abstract psychological states (Class 3). This intermediate status appears to impose greater cognitive demands on L2 learners, as Class 2 requires the integration of both physical and psychological dimensions of meaning. Learners must map syntactic structures onto semantic representations that are less transparent than those of Class 1, yet not so abstract as to trigger explicit restructuring as Class 3 might.

The chi-square results for Class 2 items further illuminate this intermediate status by revealing a gradient of NP attachment rates within the category itself. The six Class 2 items were not homogeneous in their attachment patterns (see Table 5). One item (*wakuwaku*) exhibited an NP attachment rate of 69.32%, showing a significant preference for NP attachment (*p* < 0.001). Three items (*ukiuki*, *sabasaba*, and *mukamuka*) showed attachment rates between 51.32% and 55.95%, which were close to the 50% chance level and did not reach significance (*p* > 0.05), indicating no clear preference for either NP attachment or NP suppression. One item (*uzuuzu*) had an attachment rate of exactly 50.00%, reflecting a complete lack of preference. The remaining item (*kurakura*) had an attachment rate of only 35.80%, showing a significant preference for NP suppression (*p* < 0.05), a pattern more typical of Class 3 items. This internal gradient suggests that the boundary between Class 2 and Class 3 is not categorical but gradual, with some Class 2 items already exhibiting attachment patterns that resemble those of Class 3.

Thus, the chi-square results for Class 2 not only complement the logistic regression finding that Class 2 is the most sensitive predictor of learners’ overall NP attachment preference, but also provide a finer-grained picture of how overgeneralization operates within an intermediate category. L2 learners may initially acquire the NP attachment rule for semantically transparent Class 2 items and then overextend it to less transparent ones. Consequently, performance on Class 2, which reflects the successful integration of multiple meaning dimensions, emerges as a particularly sensitive indicator of learners’ developing syntactic competence. In other words, learners who can correctly process Class 2 have likely begun the cognitive work of differentiating related syntactic rules, a prerequisite for suppressing overgeneralization to Class 3.

#### 6.2.2 Overgeneralization as a developmental phenomenon

While 6.2.1 focused on the category-specific patterns of overgeneralization, the present section examines whether these patterns are systematically linked to learners’ broader grammatical competence. The multiple regression analysis provided additional evidence that overgeneralization is not an isolated lexical phenomenon but is systematically linked to learners’ broader grammatical competence. The classification tree analysis further identified psychomime type as the primary factor distinguishing learners’ NP attachment preferences, with Class 1 forming a distinct cluster. The persistent use of NP attachment among middle- and low-proficiency learners for both Class 2 and Class 3 reflects rule overgeneralization, a phenomenon in which learners mechanically extend the NP attachment rule to inappropriate contexts.

Crucially, this overgeneralization was not uniform across learners. The classification tree further identified proficiency as a secondary factor for Class 2 and Class 3, with only high-proficiency learners demonstrating the ability to suppress the overgeneralized NP attachment rule. Middle- and low-proficiency learners, by contrast, continued to rely on NP attachment for both Class 2 and Class 3. This finding is consistent with the U-shaped developmental model (Kellerman, 1985; McLaughlin, 1990; Shirai, 1990; King, 2006), which proposes that learners do not develop NP attachment sensitivity gradually and uniformly with each incremental increase in proficiency. Rather, a qualitative shift appears to occur once a critical developmental threshold is crossed.

This threshold effect can also be understood through learners’ processing constraints (Pienemann, 1998, 2005), as grammatical operations become accessible in a hierarchical sequence, and overgeneralization can emerge only once learners have attained the developmental stage that affords the relevant syntactic-mapping operations. In the present study, suppressing NP attachment requires a more complex operation than producing it. Middle- and low-proficiency learners had likely mastered the simpler operation of producing NP attachment but not the more complex operation of suppressing it, leading them to overgeneralize the NP attachment rule. High-proficiency learners, by contrast, had reached the higher developmental stage and were able to suppress the overgeneralized rule. This is consistent with the U-shaped developmental model described above. Overgeneralization is thus not a sign of failure but a natural consequence of the hierarchical nature of grammatical development. Beyond processing constraints, the resolution of overgeneralization may also be linked to learners’ analytical ability. In the present study, L2 proficiency served as a proxy for analytical ability. This finding is broadly consistent with DeKeyser’s (2000) claim that analytical ability is a necessary condition for mapping refinement. That is, learners with greater analytical resources, as reflected in higher L2 proficiency, were better able to refine overly broad form-meaning mappings and constrain overgeneralization, whereas those with limited analytical resources remained at the U-shaped dip.

This interpretation is supported by the logistic regression results. The logistic regression analysis revealed that proficiency, when entered as a continuous variable, did not significantly predict learners’ NP+ preference (*B* = −0.05, *ns*), indicating that there is no simple linear relationship between proficiency and NP attachment preference. This discrepancy arises because the two analyses test different aspects of the relationship. The classification tree captures non-linear threshold effects by recursively partitioning the data at critical cut points, whereas the logistic regression tests for a linear trend across the entire range of proficiency scores. The non-significant linear effect of proficiency in the logistic regression therefore does not contradict the threshold effect identified by the classification tree. Rather, it confirms that proficiency’s influence is non-linear. Taken together, these two sets of results suggest that learners must reach a certain level of grammatical competence before a qualitative shift from overgeneralization to rule differentiation can occur. Proficiency modulates overgeneralization in a complex manner, strengthening both L2-based generalization and sensitivity to constraints, while the resolution of overgeneralization is structure-specific and depends on the interaction between proficiency, structural markedness, and the degree of L1-L2 overlap.

#### 6.2.3 The role of L1 transfer

The persistent overgeneralization among CSLJs raises the question of whether L1 transfer may be contributing to this pattern, given that L2 learners are most likely to overgeneralize rules from unmarked structures to marked structures, especially when the marked target structure corresponds to an unmarked L1 counterpart (Juffs, 1996; Matsunaga, 2005; Inagaki, 1997; Kuribara, 2004). Inagaki (1997), for instance, demonstrated that Japanese and Chinese learners’ acquisition of the English dative alternation was governed by the properties of the equivalent structure in their native language.

In Mandarin, words denoting bodily sensations (corresponding to Class 1) require an accompanying NP to specify the intended physical sensation. For example, the Chinese expression *wǒ de tóu téng* (‘my head aches’) requires the body-part NP *tóu* (‘head’); without it, the sentence would be incomplete. Japanese behaves similarly, as in *watasi wa atama ga itai* (‘my head aches’), where the NP *atama* (‘head’) is also required. Because both languages require NP attachment for Class 1 psychomimes, no markedness asymmetry exists, and learners do not overgeneralize in this category. This explains why learners across all proficiency levels performed with high accuracy on Class 1 items.

The situation is further complicated by the fact that, in Mandarin, words denoting psychological states (corresponding to Class 2 and Class 3) do not have a fixed NP attachment pattern. For example, one can say either *wǒ hěn fènnù* (‘I am very angry’) or *wǒ xīnli hěn fènnù* (‘I am very angry in my heart’), with the NP *xīnli* (‘in my heart’) being optional rather than obligatory. This flexibility means that CSLJs are not consistently exposed to a clear distinction between NP attachment and NP suppression in psychological-state expressions. As a result, they may default to NP attachment as a general strategy when processing Japanese psychomimes, even in Class 3 contexts where Japanese requires NP suppression. This tendency is further reinforced by the fact that Mandarin lacks the case-marking system found in Japanese (e.g., the NP-*ga*, NP-*no* construction), with attachment ambiguities typically resolved through semantic plausibility and word order rather than through morphological cues. L1 transfer is known to systematically shape overgeneralization patterns (Kellerman, 1983; Odlin, 1989), and CSLJs may rely on L1-derived semantic processing strategies rather than developing sensitivity to Japanese case-marking cues.

While the preceding discussion suggests that CSLJs may initially rely on L1-derived processing strategies, the present findings further indicate that L1 transfer is not a permanent constraint on L2 acquisition but rather a developmental phenomenon that diminishes with increasing proficiency. This view is consistent with recent work on the acquisition of Japanese sound-symbolic words. Iwasaki et al. (2017), for instance, present studies showing that L2 learners’ use of Japanese sound-symbolic words develops with increasing proficiency (Yoshioka, 2017), suggesting that L1-based constraints on processing may diminish as learners gain more L2 experience. According to Kellerman’s (1983) work on transferability, learners’ perception of the distance between L1 and L2 structures shapes whether they transfer L1 patterns, and this perception is itself subject to change over time. Odlin (1989) similarly emphasized that transfer is a dynamic process influenced by proficiency, with learners gradually moving away from L1-based processing strategies as their L2 competence develops.

Building on these theoretical insights, the present study extends this line of inquiry by examining a different structural domain, namely NP attachment in Japanese psychomimes. Matsunaga (2005) demonstrated that cross-linguistic differences in overgeneralization patterns can persist even among upper-proficiency learners, indicating that L1 transfer effects may be particularly resistant to elimination for certain marked structures. The present study reveals a complementary pattern. For this particular structure, high-proficiency learners were able to overcome L1 transfer and correctly suppress NP attachment in Class 3 contexts. This finding suggests that the persistence of L1 transfer effects may be structure-specific. While certain marked structures remain resistant to elimination even at advanced proficiency levels, others may become amenable to restructuring once learners develop sufficient processing capacity.

## 7 Conclusion and pedagogical implications

In sum, overgeneralization was widely observed in the acquisition of Japanese psychomime constructions, and it is not random but systematically constrained by the structural and semantic properties of psychomime categories.

L2 learners extended NP attachment rules to Class 3 contexts where Japanese requires NP suppression. This overgeneralization was shaped by both L1 transfer and L2 proficiency. Learners initially relied on L1-derived processing strategies due to properties of Mandarin, yet this reliance diminished with increasing proficiency, indicating that L1 transfer is a dynamic rather than static constraint. The intermediate status of Class 2, situated between concrete bodily sensation and abstract psychological states, imposed the greatest cognitive demands, producing a threshold effect: only high-proficiency learners were able to suppress the overgeneralized NP attachment rule, whereas middle- and low-proficiency learners continued to overgeneralize. These findings collectively demonstrate that the resolution of overgeneralization is constrained by structural properties and depends on how proficiency, markedness, and L1-L2 overlap interact.

Beyond these specific findings, the present study contributes to a more general understanding of L2 development. By tracing how L2 learners acquire and differentiate NP-related syntactic rules across perceptually concrete and abstract psychomime meanings, this study further elucidates the dynamic interaction between syntactic form and semantic interpretation at the syntax–semantics interface. This study suggests a possible mechanism in which grammatical rules acquired early tend to be overgeneralized, and suppressing such nontargeting patterns requires continuous improvement in learners’ grammatical competence. These results not only explain L2 learners’ differentiated performance on Japanese sound-symbolic words but also provide evidence for SLA principles concerning the incremental acquisition and restructuring of lexical-syntactic knowledge in L2 development.

These findings carry direct implications for L2 instruction, particularly in the teaching of Japanese psychomime constructions. One key implication is that explicit instruction on the syntactic differences among psychomime categories may be beneficial for L2 learners. With respect to the role of explicit knowledge, the present study supports the weak interface position (Ellis, 1993, 2006), which holds that explicit knowledge can facilitate the development of implicit knowledge by priming noticing and cognitive comparison (Schmidt, 1990). Given that learners tended to overgeneralize the NP attachment rule to Class 3, instructors should draw learners’ attention to the fact that not all psychomimes follow the same syntactic patterns. Teaching should emphasize the lexical-syntactic distinctions among psychomime categories, particularly between Class 2 and Class 3, to help learners overcome the tendency to treat them as a unified category. For example, teachers could provide minimal pairs in which the same syntactic frame is used with a Class 1 item and a Class 3 item, and ask learners to identify which NP is obligatory and which is optional.

While contrast exercises between Class 1 and Class 3 can highlight the attachment/suppression distinction, Class 2 psychomimes, which occupy an intermediate position between concrete bodily sensation and abstract psychological states, may serve as a particularly useful diagnostic category. The present study found that performance on Class 2 items was the strongest predictor of learners’ overall NP attachment preference, suggesting that accuracy on this category can indicate whether learners have begun to differentiate the syntactic rules governing NP attachment and suppression. Teachers could use learners’ performance on Class 2 items as a rough diagnostic tool to identify those who may need additional support in mastering the distinction between NP attachment and NP suppression. For learners who perform well on Class 2 items, instruction can move toward the more challenging Class 3 suppression rule; for those who struggle, additional practice with Class 2 items may help consolidate the foundational attachment rule before moving on.

Ellis (2006) also emphasized that corrective feedback is important for grammar learning and that a mixture of implicit and explicit feedback types is likely to be most effective. The present findings suggest that corrective feedback may be particularly valuable for addressing the overgeneralization observed in Class 3. Given that middle- and low-proficiency learners persistently extended the NP attachment rule to contexts where Japanese requires NP suppression, teachers should provide corrective feedback when such overgeneralization occurs in communicative contexts.

Regarding the timing of grammar instruction, Ellis (2006) suggested that grammar is best taught to learners who have already acquired some ability to use the language, rather than to complete beginners. This recommendation aligns closely with the threshold effect observed in the present study. This finding suggests that instruction targeting the NP attachment/suppression distinction may be most effective for learners who have already reached a certain developmental level. For learners below this threshold, intensive input exposure and comprehension-based practice may be more appropriate than explicit rule instruction, as they may not yet have reached the developmental stage at which the relevant syntactic-mapping operations become accessible (Pienemann, 1998).

These pedagogical implications point to the importance of a differentiated approach to grammar instruction. The non-linear nature of the proficiency effect suggests that repeated exposure to contrasting examples of NP attachment and NP suppression across different psychomime categories may help learners restructure their interlanguage grammar. Instructors could design consciousness-raising tasks that require learners to compare and contrast the syntactic behaviors of different psychomime categories, thereby promoting the gradual differentiation of overgeneralized rules. Such tasks would be consistent with the weak interface position and the use of explicit knowledge to prime noticing and cognitive comparison, as advocated by Ellis (2006).

## 8 Limitations and future directions

The present study has several limitations that warrant acknowledgment. First, the JNS participants in this study were recruited from a single university in the Tokai region. While dialectal variation is not typically considered a critical variable in the study of syntactic attachment preferences, we acknowledge that it could potentially influence naturalness judgments. Future research with JNS participants from diverse dialectal backgrounds would help confirm the generalizability of the observed patterns.

Second, the study was conducted solely with Chinese-speaking L2 learners; whether learners from other L1 backgrounds would show similar patterns remains unclear, as L1 transfer effects may differentially influence sensitivity to NP attachment constraints. Future research with learners from typologically diverse L1 backgrounds (e.g., Korean, English) is therefore necessary to determine the generalizability of the present findings.

Third, the paper-based judgment task may not fully capture learners’ performance in natural communication, as it may reflect explicit knowledge rather than automatic online processing. Future research could employ production tasks, self-paced reading, or eye-tracking.

Fourth, the stimulus set was limited to 18 psychomimes, and other lexical properties (e.g., semantic transparency, collocational strength) were not controlled. Future research should include a larger and more diverse set of psychomimes and systematically examine these lexical properties.

Fifth, the cross-sectional design captures only a snapshot of interlanguage at a single time point and cannot reveal the dynamic trajectory of overgeneralization in individual learners. Longitudinal studies tracking the same cohort over time would help clarify how NP attachment biases emerge, stabilize, or diminish with increasing L2 proficiency. Addressing these limitations in future research would strengthen the robustness and generalizability of the findings.

Compared with general vocabulary, sound-symbolic words remain an underexplored area in current linguistic research. Despite the limitations noted above, the present study provides empirical data and theoretical perspectives that may inform future investigations of these words and overgeneralization in L2 acquisition.

## Data Availability

The raw data supporting the conclusions of this article will be made available by the authors, without undue reservation.

## Footnotes

1 The JLPT (Japanese Language Proficiency Test) is currently the largest and most authoritative Japanese Language Proficiency Test system worldwide. It consists of 5 levels (N1, N2, N3, N4, and N5), with N1 as the highest level. It aims to assess and certify the Japanese language proficiency of nonnative speakers both in Japan and overseas.
